# Early Event-Related Potential During Figure and Object Perception of Abacus Mental Calculation Training Children: A Randomized Controlled Trial

**DOI:** 10.3389/fnbeh.2022.823068

**Published:** 2022-03-07

**Authors:** Dong Wang, Kongmei Zhu, Jiacheng Cui, Jianglin Wen

**Affiliations:** ^1^Department of Clinical Psychology, Beijing Chao-Yang Hospital, Capital Medical University, Beijing, China; ^2^The Third Hospital of Chaoyang District, Beijing, China; ^3^Department of Applied Psychology, Binzhou Medical University, Yantai, China

**Keywords:** event-related potential, abacus mental calculation, figure perception, object perception, children, cognitive capacity

## Abstract

The aim of this study was to discuss the effect of abacus mental calculation (AMC) on the early processing of children’s perception on numbers and objects. We designed a randomized controlled trial, and a total of 28 subjects were randomly distributed into two groups of equal numbers, namely, one group that received AMC training (training group) and the other group that did not receive training (non-training group). The subjects were asked to determine the figures and objects shown on the computer screen and were recorded on the computer. The event-related potential (ERP) component (N1, N170, P1, and P2) of different brain areas between the two subject groups was compared. Compared with the non-training group, the training group’s P1 in the occipital region showed a larger amplitude and a longer potential period. For N1, the training group showed a longer potential period. Additionally, for N170, the training group showed a smaller amplitude. Finally, the observation of P2 showed a smaller amplitude in the training group and a longer potential period in the condition of object stimulus. Overall, the activated degree of the occipital region of children who received AMC training was enhanced, while the activated degree of the central region of the forehead and temporal occipital region was slightly down. Meanwhile, the potential periods of all components were extended. Therefore, long-term AMC training can change children’s cortical function activities.

## Introduction

Abacus mental calculation (AMC) is a cognitive skill based on abacus’ use to form bead-image movement in the brain through actual bead-driven training, simulated bead-driven training, and image bead-driven training. It is an advanced calculation function of the brain. AMC training can make a person solve mathematical problems more accurately and quickly (Hu et al., 2011). There are three stages to acquire this capability: first, one should learn to calculate using a real abacus (a simple device consisting of beads and rods); second, after becoming familiar with the operation of the abacus, he/she will be instructed to imagine moving the beads in his/her mind with actual finger movements to finish the calculation; and third, he/she can try to calculate *via* the imaginary abacus completely. AMC can improve many aspects of cognition (Hanakawa et al., 2003). Previous studies have found that AMC can not only improve the efficiency of mental calculation of children but also enhance children’s attention, memory, thinking, and various basic cognitive abilities (Hatta and Miyazaki, 1989; Liu and Sun, 2017). In addition to cognitive improvements, AMC training was also found to improve activation levels and neuroplasticity in some brain regions (Czigler and Csibra, 1990; Stiles, 2000; Tanaka et al., 2012; Weng et al., 2017) can also be found. Conversely, some studies have found that AMC training cannot improve cognitive abilities. A study by Barner et al. (2016) involving 183 children over 3 years reported that AMC training provided no benefit for basic cognitive abilities. A study by Xie et al. (2018) assessing 162 children for 1 year supported the finding that abacus arithmetic had a slight effect on children’s memory only in the early stages of training, while no significant difference was observed in the memory performance between the training group and the non-training group after training. So, the influence of AMC on cognition should be further explored. Perception is considered as a basis for the superior cognitive stage and regulates the relationship between the body, the environment, and related behaviors (Dong and Bao, 2021). This study aims to adopt event-related potential (ERP) technology to investigate the impact of AMC on the early processing of children’s perception on numbers and objects from the angle of sensory perception.

We hypothesized that the training group was able to process digital stimuli with less brain activation, which was represented by a reduction in the amplitude of the ERP component. In addition, AMC training can improve children’s early attention to visual stimulus, which is manifested by a longer potential period of ERP components.

## Materials and Methods

### The Objects of Study

The experiment was designed with a randomized control trial. The samples were randomly selected from students of grades 3 and 4 at Bei Guan Central Primary School in Weifang from March to April 2020. A total of 28 students (14 boys and 14 girls) participated in the study. They were randomly divided into two groups, namely, 14 children who received AMC training (training group) (starting from the first grade of primary school, two 50-min sessions a week for 20 weeks) and other 14 children who did not receive training (non-training group); there were 7 boys and 7 girls in both groups. The participants aged 10–11 years, with an average age of 10.5 years, and the average age was 10.4 years in the training group, whereas it was 10.6 years in the non-training group. There were no significant differences in age, family background, educational background, and score between the two groups. The students participated in this ERP experiment for the first time. Since the difference in handedness might lead to different active patterns of the brain, we selected only right-handed participants to make the ERP’s result comparable. In addition, all participants had visual acuity or corrected visual acuity of 1.0 or higher. This study was approved by the Ethics Committee of Beijing Chaoyang Hospital, and all subjects have signed informed consent.

### Experimental Instrument

A 64-channel NeuroScan ERP workstation made in the United States was used. The electrodes were set according to the international 10/20 system standard (Xu et al., 2012). The reference electrodes were placed at the right and left mastoids, and the ground electrode was placed 1 cm under the forehead hairline. At the same time, both horizontal and vertical electrooculography (EOGs) were recorded. Filtering bandpass was 0.05–40 Hz. The sampling rate was 1,000 HZ/channel, and the scalp resistance was less than 5 kΩ. A standard 32-channel electrode cap for electroencephalogram (EEG) was used to record the electrical activity of the brain.

### Materials for the Simulated and Experimental Procedure

**FIGURE 1:**
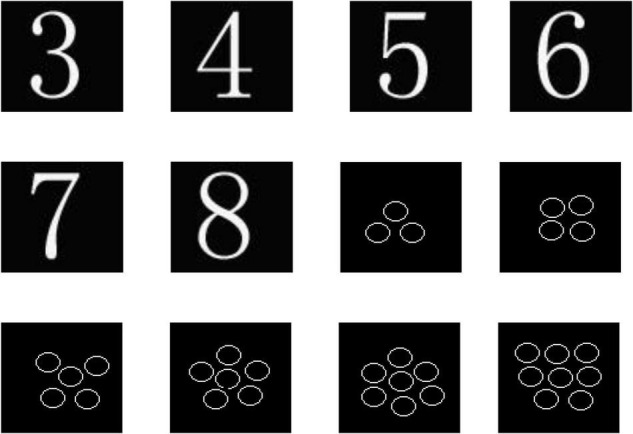
Pictures of number and object-controlled experiments.

The stimulation consisted of two types. The first type of stimulation was a number; 3–8 numbers were randomly arranged and individually shown on the computer screen. The second type of stimulation was composed of circle-representative objects, numbering 3–8 (Figure 1). Stim2 was used to write a stimulus program. The stimulation was randomly arranged. Each stimulus was shown for 500 ms with a stimulus interval (ISI) of 500 ms. The stimulus was repeated 10 times for a total of 120 times. The stimulation was shown on a 15-foot computer screen. The background was black, and the numbers or circles were white. The subjects of the experiment sat on chairs, facing the display screen. The sight distance was 60 cm. At the start of the experiment, the subjects were required to look at the point of fixation (a cross in white). When the stimulus appeared, the students were told to pay more attention to observing the shown numbers and pictures. When the numbers appeared, the students were asked to press the digit 1 button on the key box, while the object appeared, they were asked to press the digit 2 button. To eliminate the influence of left- and right-hand buttons on the computer, the selection of buttons was cross-balanced among the students.

During the experiment, the focus was on the changes in EEG. The experiment results were saved once the experiment was completed. EEG records were then added to calculate the average. The reference values of right and left mastoids were converted to the average reference voltage to make corrections, excluding wink, eye movement, EMG, and other artifacts. The analytic window of EEG was −100–500 ms, and −100–0 ms was used as the baseline to make corrections. Through classification and addition, two ERPs were caused by different stimuli, and two types of ERPs were caused by different subjects. Given the purpose of this experiment, several electrode points in the temporal occipital region (P7/P8), the occipital region (O1, OZ, and O2), and the central region of the forehead (FZ, FCZ, and CZ) were used as the representative points in the analytic position. The analysis time window of P100 at the real scalp was 50–150 ms; the analysis time window of N170 was 140–220 ms; the analysis time window of P2 at the fore scale was 180–270 ms; and the analysis time window of N1 was 75–150 ms. The measurement methods in peak amplitude and peak potential periods were used. The results were filtered using the zero phase with a bandwidth of 0.8–30 Hz.

## Statistical Analyses

The SPSS statistical software was used in the 3-factor analysis of variance on the aforementioned amplitudes and potential periods. The between-subject factor refers to the type of subject (two levels, i.e., the training group and the non-training group). The within-subject factor refers to the type of stimulus (two levels: figure and circle) and the positions of electrodes (rear P1: three levels; N170: two levels; and front N1, P2: three levels). We used the SPSS17.0 to analyze and process the data. The Greenhouse-Geisser method was used to correct the *P-*value.

## Results

### Features of Event-Related Potentials’ Early Component

**FIGURE 2:**
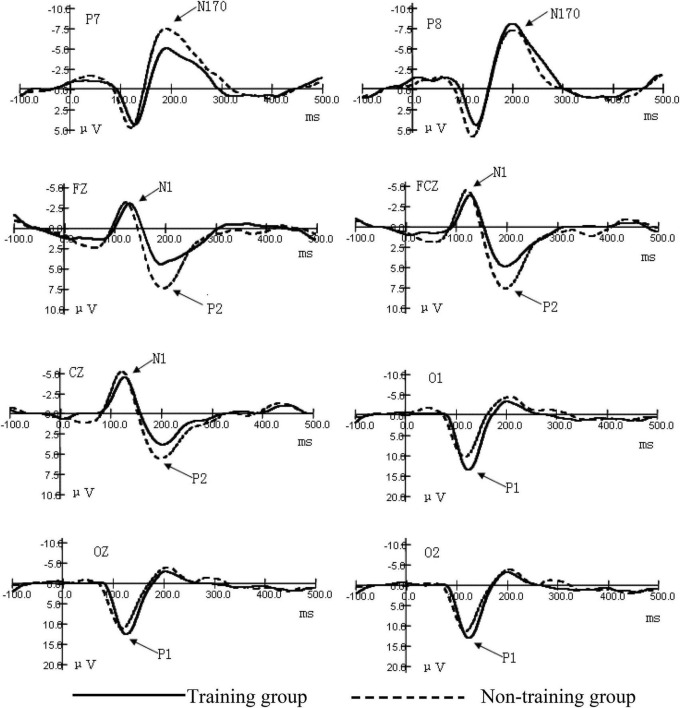
Grand average ERP waveforms for the early components of figures.

**FIGURE 3:**
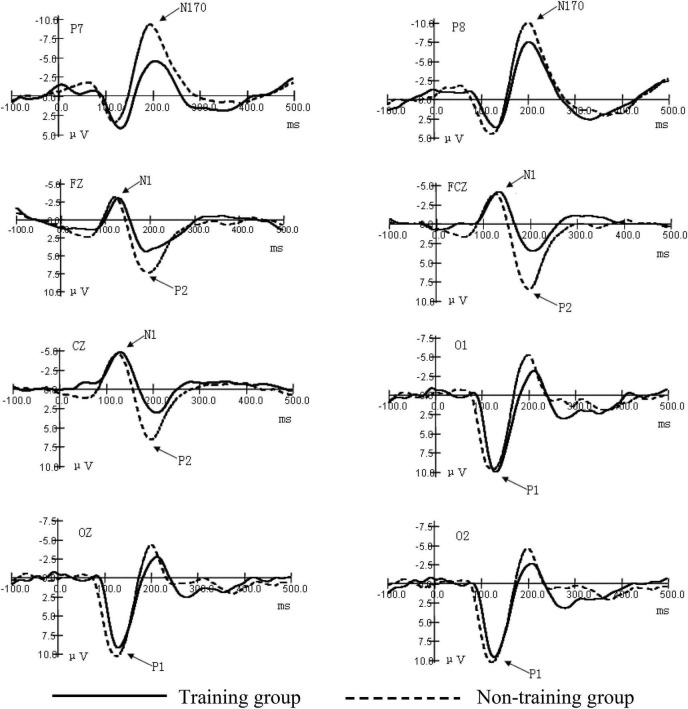
Grand average ERP waveforms for the early components of objects.

**FIGURE 4:**
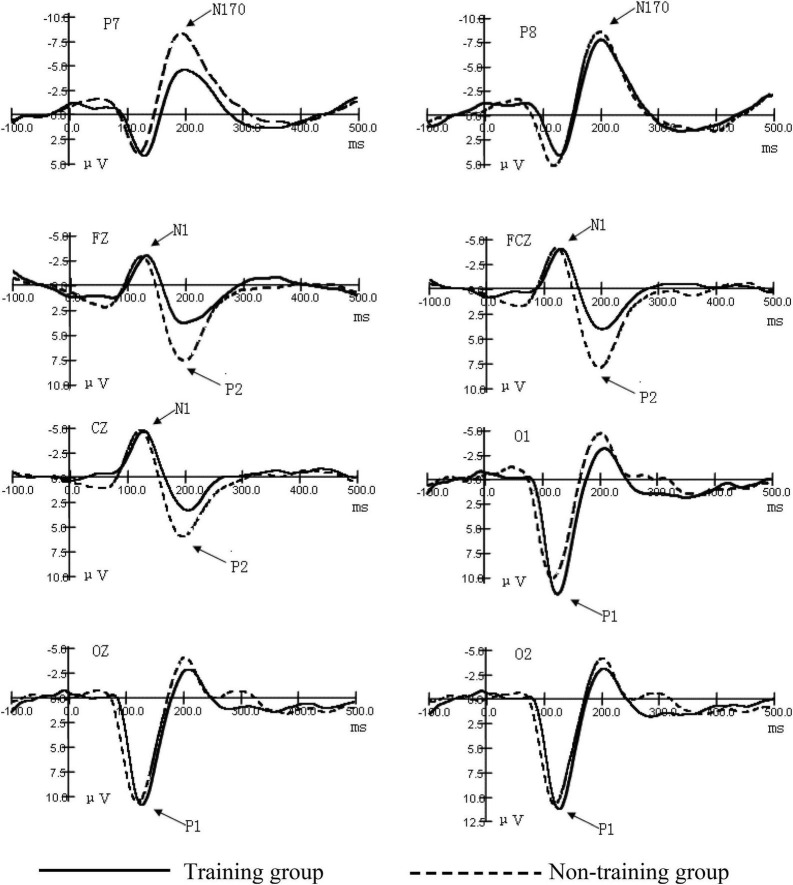
Grand average ERP waveforms for the early components of figures and objects.

As shown in Figures 2–4 (ms refers to millisecond and μV refers to microvolt), the ERPs of the two groups caused by different stimuli evoke consistency on the basic features. A general visual-evoked response can be observed, i.e., P1 mainly composed of the occipital region (O1, OZ, and O2); N1 and P2 were in the forehead central area (FZ, FCZ, and CZ); N170 was in the temporal occipital region (P7 and P8). However, when the two groups were compared, significant differences in the amplitudes were observed. The P1 amplitude of the training group was larger than the non-training group, especially the P1 amplitudes of the training group caused by the figure was larger than that of the non-training group, while in each group, the difference of P1 amplitudes caused by object stimulus was not significant. The P1 potential period of the training group was longer than that of the non-training group. No significant difference existed between the N1 amplitude of the training group and that of the non-training group; the N1 potential period of the training group was longer than that of the non-training group. The N170 amplitudes of the training group were smaller than those of the non-training group, especially in the P7 electrode position; the N170 potential period of the training group was longer than that of the non-training group. The P2 amplitude of the training group was smaller than that of the non-training group; the P2 potential period of the training group was longer than that of the non-training group, especially under the condition of object stimulus.

### Comparison of the Occipital Region P1 Amplitudes and the Potential Periods Between the Two Groups Under Different Stimulation Conditions

The interaction between the positions of the electrodes of occipital region P1 amplitudes and the types of subjects was significant (*F* = 12.69, *P* < 0.01). After the interaction of the two factors was found, we subsequently conducted a simple effect analysis. We hoped to further analyze the different levels of factors that have a significant effect at a certain level of another factor. Further simple effect analysis showed that the main effect in the type of subject was significant in the left-brain area (O1) (*F* = 5.36, *P* < 0.05). This indicates that the training group has an impact on the amplitude of the P1 component, and this effect is significant at the O1 electrode position. The P1 amplitudes of the training group were larger than those of the non-training group. The main effect in the type of stimulus was significant (*F* = 49.85, *P* < 0.01). The interaction between the type of stimulus and the type of subject was significant (*F* = 30.35, *P* < 0.01). Further simple effect analysis showed that, under the condition of numerical stimulus, the main effect of the type of subject was significant (*F* = 4.85, *P* < 0.05). The occipital region P1 amplitudes of the training group were larger than those of the non-training group. However, under the condition of circle stimulus, the main effect of the type of subject was not significant. These results showed that the P1 amplitudes were significantly greater in the training group than in the non-training group under visual forms of digital stimuli. From the potential period, the main effect of the type of stimulus in the occipital region P1 potential period was significant (*F* = 8.538, *P* < 0.01). The main effect of the type of subject was significant (*F* = 15.488, *P* < 0.01). The P1 potential period of the training group was longer than that of the non-training group, The results indicate that AMC training can prolong the potential period of P1 (Table 1).

**TABLE 1:** Comparison of occipital P1 (μV) amplitude and potential period (ms) between the two groups (*n* = 14, $\bar{x}\pm s$) under the conditions of different stimuli.

Electrode	Group	Object stimulus		Figure stimulus	
		Amplitude (μV)	Potential period (ms)	Amplitude (μV)	Potential period (ms)
O1	Training group	10.90 ± 4.19	132.57 ± 11.69	14.28 ± 4.52	126.43 ± 7.34
	Non-training group	8.50 ± 3.59	124.14 ± 13.42	10.35 ± 3.22	116.5 ± 5.23
OZ	Training group	10.54 ± 4.59	132.50 ± 11.75	13.07 ± 3.67	126.21 ± 7.85
	Non-training group	11.14 ± 3.52	116.78 ± 6.09	10.863.17	116.79 ± 6.10
O2	Training group	10.72 ± 2.83	131.07 ± 12.9	13.62 ± 4.26	127.43 ± 5.83
	Non-training group	10.69 ± 5.01	124.14 ± 10.69	10.83 ± 3.03	117.14 ± 5.08

*$\bar{x}$ ± s (mean ± SD): within the range represents a large probability event and outside the range represents a small probability event.*

### Comparison in the Central Region N1 Amplitude and the Potential Period Between the Two Groups

The significant effect of forehead central region N1 amplitude was only reflected in the position of the electrode (*F* = 23.13, *P* < 0.01). Analysis of the N1 potential period showed that the main effect of the position of the electrode was significant (*F* = 4.72, *P* < 0.05). The main effect of the type of subject was significant (*F* = 13.37, *P* < 0.01). To sum up, the N1 potential period of the training group was longer than that of the non-training group (Table 2).

**TABLE 2:** Comparison of forehead central region N1 amplitude (μV) and potential period (ms) between the two groups (*n* = 14, $\bar{x}\pm s$).

Electrode	Group	Amplitude (μV)	Potential period (ms)
FZ	Training group	3.64 ± 1.59	133.07 ± 8.29
	Non-training group	3.43 ± 3.16	123.07 ± 6.99
FCZ	Training group	4.74 ± 1.89	132.21 ± 7.72
	Non-training group	4.54 ± 3.12	121.54 ± 5.88
CZ	Training group	5.42 ± 2.44	129.25 ± 8.20
	Non-training group	5.17 ± 2.54	121.96 ± 5.46

### Comparison of the Temporal Occipital Region N170 Amplitude and the Potential Period Between the Two Groups

The position of the electrode on the temporal occipital region N170 amplitude has a significant main effect (*F* = 4.69, *P* < 0.05). The interaction between the position of the electrode and the type of subject was significant (*F* = 3.91, *P* < 0.05). Further simple analysis showed that in the P7 electrode position, the main effect of the type of subject was significant (*F* = 12.98, *P* < 0.01). The analysis shows that the AMC training has a significant effect on the N170 amplitudes in the P7 position. The AMC training significantly reduced the N170 amplitudes in the temporal regions, especially when stimulated by numbers. The main effect of the type of subject was significant (*F* = 4.97, *P* < 0.05), which shows that, in general, the N170 amplitudes of the training group were smaller than those of the non-training group. The analysis of the N170 potential period showed that the main effect of the subject was near significant (*F* = 3.83, *P* = 0.061). The N170 mean potential period of the training group was longer than that of the non-training group. These suggest that AMC training prolongs the N170 potential period in children (Table 3).

**TABLE 3:** Comparison of the temporal occipital region amplitude (μV) and potential period (ms) between the two groups (*n* = 14, $\bar{x}\pm s$).

Electrode	Group	Amplitude (μV)	Potential period (ms)
P7	Training group	4.87 ± 3.38	199.64 ± 11.87
	Non-training group	9.57 ± 3.51	189.07 ± 14.18
P8	Training group	7.50 ± 5.01	199.25 ± 8.43
	Non-training group	9.69 ± 5.38	194.67 ± 11.71

### Comparison of the Central Region of the Forehead P2 Amplitude and the Potential Period Between the Two Groups

The main effect of the position of the electrode of P2 amplitude was significant (*F* = 11.05, *P* < 0.01). The main effect of the type of subject was significant (*F* = 12.22, *P* < 0.01). The P2 amplitudes of the training group were smaller than those of the non-training group. The analysis of the potential period showed that the main effect of the type of subject was significant (*F* = 4.76, *P* < 0.05). The P2 potential periods of the training group were longer than those of the non-training group. The interaction between the type of stimulus and the type of subject was significant (*F* = 7.29, *P* < 0.05). Further simple analysis showed that, under the condition of object stimulus, the main effect of the type of subject was significant (*F* = 8.41, *P* < 0.01), which indicates that the AMC training has a significant difference in the amplitudes of P2 induced by the digital stimulus. The P2 potential periods of the training group were longer than those of the non-training group. Under the condition of figure stimulus, the main effect of the type of subject was not significant (Table 4).

**TABLE 4:** Comparison of the central region of the forehead P2 amplitude and the potential period between the two groups under different stimulation conditions (*n* = 14, $\bar{x}\pm s$).

Electrode	Group	Object stimulus		Figure stimulus	
		Amplitude (μV)	Potential period (ms)	Amplitude (μV)	Potential period (ms)
FZ	Training group	4.15 ± 3.58	214.00 ± 16.71	4.95 ± 2.65	207.85 ± 25.31
	Non-training group	8.68 ± 3.95	194.91 ± 10.54	7.80 ± 2.79	201.85 ± 13.23
FCZ	Training group	4.15 ± 3.60	212.71 ± 16.84	5.00 ± 2.79	200.92 ± 18.69
	Non-training group	8.84 ± 3.34	194.42 ± 8.83	8.06 ± 2.30	199.92 ± 12.85
CZ	Training group	3.55 ± 3.15	211.92 ± 16.67	4.00 ± 2.62	207.07 ± 19.95
	Non-training group	6.74 ± 2.76	197.07 ± 16.44	6.67 ± 1.83	206.14 ± 23.55

## Discussion

This is the first study that investigates the effect of AMC on the early process stage of cognition. Our objective was to investigate the effects of AMC training on the early cognitive stage of children. To this end, we used the visual stimulus recognition task, using two different forms of stimulus (number and object), to observe the differences in ERP components between the training and non-training groups. Finally, we compared the amplitudes and potential periods of components. Previous studies have focused on other aspects of cognition, such as working memory, learning ability, mathematical computation, and intelligence, whereas this study is the first to focus on the impact of AMC on early cognitive processing, namely, perception and attention. The results show that a significant difference in the ERP early component of figure perception and circle-object perception exists between the training group and the non-training group and partially proves our hypothesis. The ERP early component reflects the mental process of stimulus discrimination (Simson et al., 1985; Hillyard and Anllo-Vento, 1998). Therefore, this experiment shows that AMC has a significant impact on the early process stage of numbers and objects (particularly numbers), i.e., the sensory perception process stage. The ERP amplitude always reflects the disbursement of the psychological resources as processing information. The amplitude is positively related to the number or strength of neurons activated (Hillyard et al., 1998; Wang et al., 2003). Under the condition of number stimulus, the occipital region P1 amplitudes of the training group are larger than those of the non-training group, which indicates that AMC training enhances the early activation of children to visual information processing. Some studies on brain plasticity show that AMC can enhance the activated degree of some functional regions in the cortex and reorganize the cortex (Hatta and Miyazaki, 1989; Hillyard and Anllo-Vento, 1998). In previous studies, changes in the function of the occipital lobe, parietal lobe, and circuits between these regions were mainly found (Belkacem et al., 2020). We deduce that in brain plasticity, AMC training has a certain influence on the occipital cortex function of children, and this is consistent with previous studies.

Furthermore, this study has found that a significant difference in the N170 component caused by stimulus in the temporal lobes of both sides between the training group and the non-training group exists. N170 is always deemed as the specific component for face recognition (Wang et al., 2019). However, some studies point out that N170 can also reflect visual processing of object discrimination and classification (Rousselet et al., 2004; Caharel and Rossion, 2021). Whether the stimulus is a number or an object, N170 amplitudes of non-mental abacus children are larger than those of mental abacus children, indicating that mental abacus children consume less brain resources than non-mental abacus children as discriminating stimulus.

The component P2 in the central region of the forehead not only reflects the visual coding stage of sensory perception but also relates to the early activation of figure cognitive processing (Kong et al., 1999). In the experiment, P2 amplitudes of the training group are significantly lower than those of the non-training group, indicating that children trained in abacus arithmetic were more likely to have their cognitive processing of numbers activated. The training method of AMC (numbers are converted to imaged beads in the brain) is closely related to figure processing (Du et al., 2014). Early studies have revealed that number processing is automatic, which means that this process begins immediately and involuntarily upon seeing numbers (Tanaka et al., 2012; Yao et al., 2015), so long-term training may reduce the early activation threshold of children to numbers and improve their automatic degree of cognitive processing of figures. Experiment results show that rear P1, N170, and front N1 and P2 have different potential periods among subjects, indicating that in case of no task, AMC training will extend children’s perception time to stimulus even if it can enhance children’s early sensory perception and improve attention.

Studies on brain plasticity show that learning and training can change the activation status of different brain areas (Kasahara et al., 2013; Zhou et al., 2020). The findings of this study are consistent with the existing research results.

This study provides a perspective to improve the cognitive ability of children and enhance our understanding of the underlying mechanism of cognitive activity. AMC training can improve children’s perceptual ability, and this might be the basis of the superior cognitive processes. We also know that AMC training can alter the activity of some specialized brain regions. Therefore, the introduction of abacus mental arithmetic training in primary education may play a positive role in children’s mathematical ability and cognitive development, and pilot education also can be carried out in some regions.

The study also has some limitations. First, the sample only has 28 objects, therefore, the general applicability of conclusions should be considered carefully. However, the experiment is a duplicate measurement trial, and it can make up for the shortage of small samples to some extent. Second, all subjects are children and the average age is only 10.5 years. Notably, cognition is developing. Whether the results that we have observed in children can be embodied in adulthood person is not sure. Finally, we focused only on some aspects of the fundamental capacity of cognition. In the future, we will further investigate the changes in cognitive dimensions associated with mental arithmetic training, while expanding the sample to adults or conducting longitudinal studies.

## Conclusion

By comparing with ordinary children, the activated degree of the occipital region of the training group is enhanced, while the activated degree of the central region of the forehead and temporal occipital region is slightly down. Meanwhile, the potential periods of all components are extended. Therefore, after long-term AMC training, children’s cortical function activities can be improved.

## Data Availability Statement

The original contributions presented in the study are included in the article/supplementary material, further inquiries can be directed to the corresponding authors.

## Ethics Statement

The studies involving human participants were reviewed and approved by the Ethics Committee of Beijing Chaoyang Hospital. Written informed consent to participate in this study was provided by the participants’ legal guardian/next of kin.

## Author Contributions

DW and KZ were mainly involved in experimental design and testing. JC was mainly involved in testing, data processing, and manuscript writing. JW was mainly involved in manuscript writing. All authors contributed to the article and approved the submitted version.

## Conflict of Interest

The authors declare that the research was conducted in the absence of any commercial or financial relationships that could be construed as a potential conflict of interest.

## Publisher’s Note

All claims expressed in this article are solely those of the authors and do not necessarily represent those of their affiliated organizations, or those of the publisher, the editors and the reviewers. Any product that may be evaluated in this article, or claim that may be made by its manufacturer, is not guaranteed or endorsed by the publisher.
